# Molecular Characterization of *Cyclospora-*like Organisms from Golden Snub-nosed Monkeys in Qinling Mountain in Shaanxi Province, Northwestern China

**DOI:** 10.1371/journal.pone.0058216

**Published:** 2013-02-28

**Authors:** Guang-Hui Zhao, Mei-Mei Cong, Qing-Qing Bian, Wen-Yu Cheng, Rong-Jun Wang, Meng Qi, Long-Xian Zhang, Qing Lin, Xing-Quan Zhu

**Affiliations:** 1 College of Veterinary Medicine, Northwest A&F University, Yangling, Shaanxi Province, China; 2 College of Animal Science and Veterinary Medicine, Henan Agricultural University, Zhengzhou, China; 3 State Key Laboratory of Veterinary Etiological Biology, Key Laboratory of Veterinary Parasitology of Gansu Province, Lanzhou Veterinary Research Institute, Chinese Academy of Agricultural Sciences, Lanzhou, Gansu Province, China; The University of Hong Kong, China

## Abstract

*Cyclospora* spp. have been identified as one of the most important intestinal pathogens causing protracted diarrhea in animals and human beings. To determine the *Cyclospora* species in the non-human primate *Rhinopithecus roxellanae*, a total of 71 fecal samples from 19 endangered snub-nosed monkeys in Shaanxi province were collected and examined using Sheater’s sugar flotation technique and by sequencing the fragments of 18S rDNA. Only two *Cyclospora* isolates from 2 golden snub-nosed monkeys (*R. roxellanae*) were obtained and identified between July 2011 and August of 2012. The sequences of the 18S rDNA for the two *Cyclospora* isolates were 477 bp, with no nucleotide variation between them. Phylogenetic analysis based on the 18S rDNA sequences revealed that the two *Cyclospora* isolates were posited into the clade *Cyclospora* spp. and sistered to *C. colobi*. These results first showed that *Cyclospora* infection occurred in *R. roxellanae* in hot and rainy weather, which would provide useful information for further understanding the molecular epidemiology of *Cyclospora* spp. and the control of *Cyclospora* infection in non-human primates as well as in human beings.

## Introduction

Coccidian *Cyclospora* is an obligate intracellular apicomplexan protozoa that inhabits in the mucosal epithelium of the intestine or bile duct of various vertebrates [1], and sometimes it also is found in invertebrates, eg. the only species *C. glomericola* in the millipede [2]. Nineteen *Cyclospora* species have been described and identified in reptiles, insectivores, snakes, rodents, primates [1], [3], [4] and humans [5]. It was 1979 when *Cyclospora* spp. was firstly detected in three patients in Papua New Guinea [5] and eventually named as *Cyclospora cayetanensis* according to the classical morphology [6]. Diarrhea has been identified as the typical clinical symptom of cyclosporiasis in humans, especially in travelers [7], [8] and AIDS patients [9], but the severity is closely related to person’s immunity [10]–[12].

In China, *Cyclospora* infection in humans has been reported in Anhui, Zhejiang and Henan provinces [13]–[15]. These studies suggested that the prevalence of *C. cayetanensis* is higher in villages than that in towns, and the prevalence of *Cyclospora* infection is associated with age and season. However, the seasonality of *Cyclospora* infection in humans is likely to be influenced by the temperature, rainfall, humidity, and other environmental factors worldwide. For example, the *Cyclospora* prevalence in humans were markedly higher during warm and rainy seasons in Guatemala [16], Jordan [17], Nepal [18], [19], Peru [20], USA and Canada [21], but in Turkey the *Cyclospora* prevalence was noted at hot and dry seasons [22], while in Haiti [11] *Cyclospora* infection occurred during the cooler and drier months of the year.

Understanding the transmission of *Cyclospora* has important implications for controlling and preventing *Cyclospora* infection in humans and animals. Though the exact transmission routs for human infection with *Cyclospora* is still a question, several studies have provided valuable information. Contacting with soil has been identified as a risk factor for *Cyclospora* infection of humans based on studies of *Cyclospora* infections in Guatemala [16], Peru [23], and USA [24] where children and farmers are frequently infected through contacting with contaminated soil directly or indirectly [25].

Zoonotic transmission also seemed to be a risk factor. Studies in Guatemala [16], Jordan [17], and Peru [20] showed that people whose fecal samples were positive for *C. cayetanensis* usually had history of feeding livestock and poultry. Using PCR approach, *C. cayetanensis* oocysts had been detected from fecal samples of both domestic and street dogs, wild chickens inhabiting the forest regions near villages, and *Macaca mulatta* rhesus monkeys that wandering through the forest region [26]. All these results suggested the zoonotic nature of this parasite, although it is yet to know whether *C. cayetanensis* can infect these copraphagic animals naturally or these animals just pass oocysts through the gut. Moreover, several studies indicated the presence of *Cyclospora*-like oocysts in chickens [27], ducks [28], dairy cattle [29], monkeys [30], [31], mice [18], rats [18] and dogs [18], [32] and zoo animals (non-human primates, carnivores, and artiodactyla) [33]. Of them, the non-human primates have the closest genetic relationships with humans. *C. cayetanensis*, *C. cercopitheci*, *C. colobi* and *C. papioni* have been found in *Macaca mulatta* rhesus monkeys, *Cercopithecus aethiops* Linnaeus, *Colobus guereza* Ruppell, *Papio anubis* Lesson [26], [34]. Therefore, determination of *Cyclospora* spp. in monkeys would have important implications for controlling *Cyclospora* infection in humans.

The golden snub-nosed monkey (*Rhinopithecus roxellanae*) is one of the endangered and precious wild animals and has been listed as Category I in the List of Key Protected Wildlife in China. It mainly distributes in Sichuan, Gansu, Shaanxi and Hubei provinces. The objective of the present study was to determine the prevalence and species of *Cyclospora* in golden snub-nosed monkeys in Shaanxi province, northwestern China.

## Materials and Methods

### Ethics Statement

The performance of this study was strictly accord to the recommendations of the Guide for the Care and Use of Laboratory Animals of the Ministry of Health, China, and our protocol was reviewed and approved by the Research Ethics Committee of Northwest A&F University. All the fecal samples were collected from animals after the permission of the Shaanxi Rare Wildlife Rescue Breeding Research Center, with no specific permits being required by the authority for the feces collection.

### Fecal Sample Collection

A total of 19 golden monkeys in Shaanxi Rare Wildlife Rescue Breeding Research Center in Shaanxi province of China were included in this survey. From July 2011 to August 2012, fresh stool samples from golden snub-nosed monkeys were collected, placed immediately in disposable plastic bags, and stored at 4°C for further detection.

### Microscopic Examination and Oocysts Sporulation

All stool samples were suspended in water, filtered by 160 mesh sieve, and centrifuged at 3000 rpm for 3 min. The sediment was concentrated by Sheater’s sugar flotation technique. Fecal specimens were examined by microscopy for the presence of *Cyclospora*-like oocysts [29], [34]–[37]. Photomicrographs of parasites were taken using a camera.

The *Cyclospora*-like organism was mixed with a 2.5% aqueous (w/v) potassium dichromate (K_2_Cr_2_O_7_) solution in a 3∶1 ratio and stored at room temperature for morphological observation of sporulated oocysts after incubation for 10 days. The positive fecal samples with *Cyclospora*-like organism were kept in 2.5% aqueous (w/v) potassium dichromate solution for molecular characterization.

### DNA Extraction and PCR Amplification of 18S rDNA

The dung samples were washed with water and the upper potessium dichromate solution was discarded. Genomic DNA was extracted from the fecal samples according to the protocols of the kit (Omega), and stored at −20°C until further use. The 18S rDNA of each DNA sample was amplified using the nested primer pairs described by Relman et al. [38] following the reaction system of Li et al. [29]. Each amplicon was examined by agarose gel electrophoresis to validate amplification efficiency.

### Sequencing of the Amplicons and Genetic Analysis

The positive PCR products were sequenced directly by Sangon Biotech (Shanghai) with internal primers described by Relman et al. [38]. The obtained sequences were aligned with 18S rDNA sequences of *Cyclospora* spp. available in GenBank™ using BLAST program. Sequence differences (*D*) among *Cyclospora* spp. were determined by pairwise comparisons using the formula *D = *1−(*M*/*L*), where *M* is the number of alignment positions at which the two sequences have a base in common, and *L* is the total number of alignment positions over which the two sequences are compared [39]–[41].

To determine the taxonomic identity of *Cyclospora* spp. isolated in the present study, phylogenetic relationships of parasites in the genus *Cyclospora* and some *Eimeria* species with high similarity to *Cyclospora* isolated herein were re-constructed using neighbor joining (NJ) method in Mega 4.0 [42] with substitution model of Kimura 2-parameter. The consensus tree was obtained after bootstrap analysis with 1000 replications, with values above 50% reported. Phylograms were drawn using the Tree View program version 1.65 [43].

## Results

**Figure 1:**
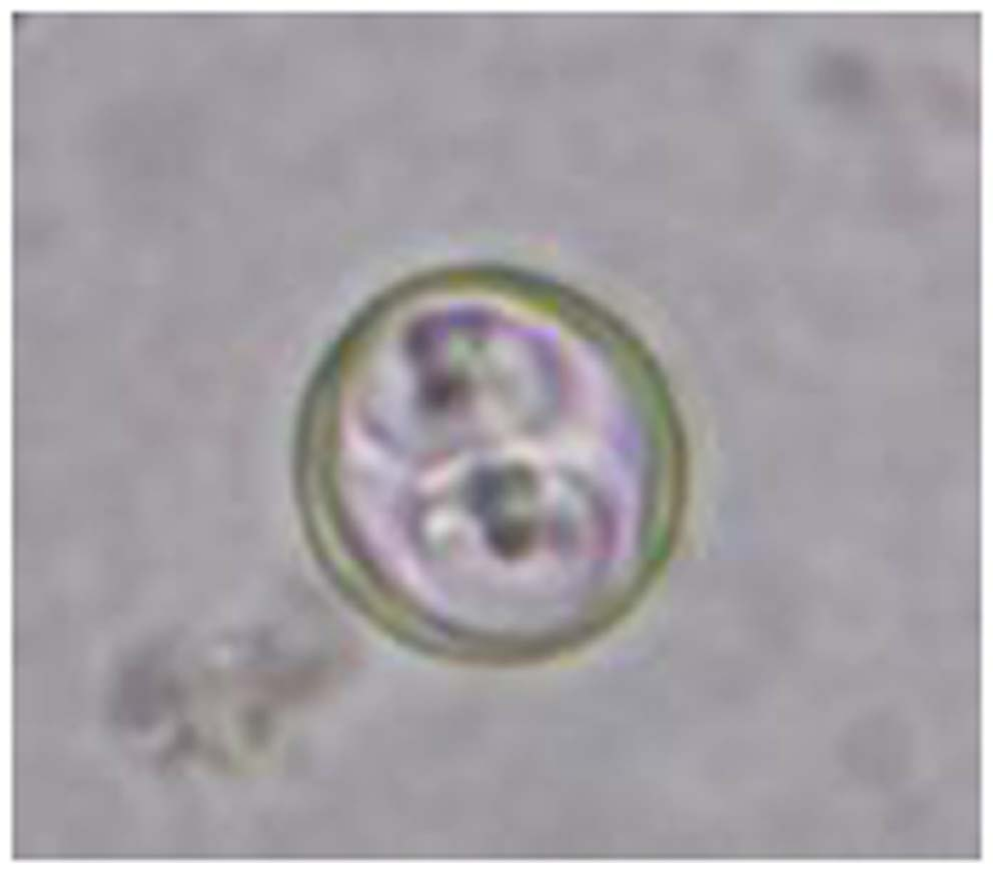
*Cyclospora*-like oocyst found in the feces (wet mount preparation) of the golden snub-nosed monkeys. Sporulated oocyst: two sporocysts were seen within one oocyst, and each sporocyst contains two sporozoites. Magnification: 400×.

Of 71 fecal samples examined from July 2011 to August 2012, only 2 samples were positive for *Cyclospora*-like oocysts by microscopy. These two samples were then transferred into potassium dichromate solution (2.5%) and incubated at room temperature for sporulation. The sporulated oocysts were spherical, with refractile “cyst-like” structure. Two sporocysts were seen within one oocyst, and each sporocyst contained two sporozoites (Figure 1).

**Figure 2:**
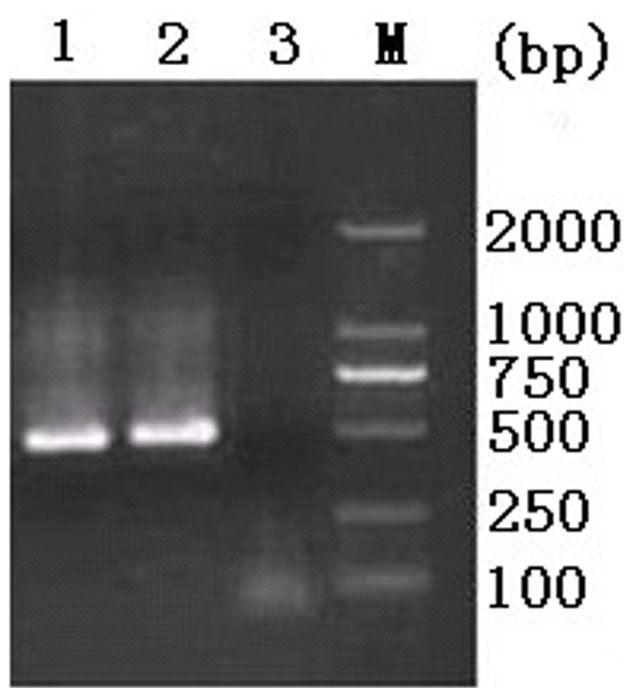
Molecular identification of *Cyclospora*-like organism in the feces of the golden snub-nosed monkeys. PCR products generated with nested eimeriid-range restricted primers; 500-bp amplicons were produced from the DNA of *Cyclospora*-like oocysts. Lane M: DL-2000 DNA Marker, lanes 1–2: oocyst specimens from golden snub-nosed monkeys 1 and 2, respectively, and lane 3: Negative control.

To determine the specific identity of *Cyclosopora*-like oocysts, genomic DNA of each isolate was extracted and used for PCR amplification of 18S rDNA. The lengths of the PCR products from both isolates were approximately 500 bp on agarose gel electrophoresis (Figure 2). Then the purified amplicons were sequenced and intra- and inter-species sequence differences in the 18S rDNA regions were determined. The two obtained sequences were identical, with 99% similarity to corresponding sequences of *Cyclospora* sp. available in GenBank™ (accession numbers AF111183, AF111184, AF1111185, AF111186 and AF111187). After removing the inaccurate sequences in the both ends, 477 bp of the 18S rDNA sequence were used for pairwise comparisons. Sequence differences between the *Cyclospora*-like isolates obtained in the present study and *C. cayetanensis*, *C. cercopitheci*, *C. colobi*, *C. papionis* were 0.8%, 1.0%, 0.4%, and 0.8%, respectively. There results suggested that the two *Cyclospora*-like isolates obtained in the present study may represent *C. colobi*.

**Figure 3:**
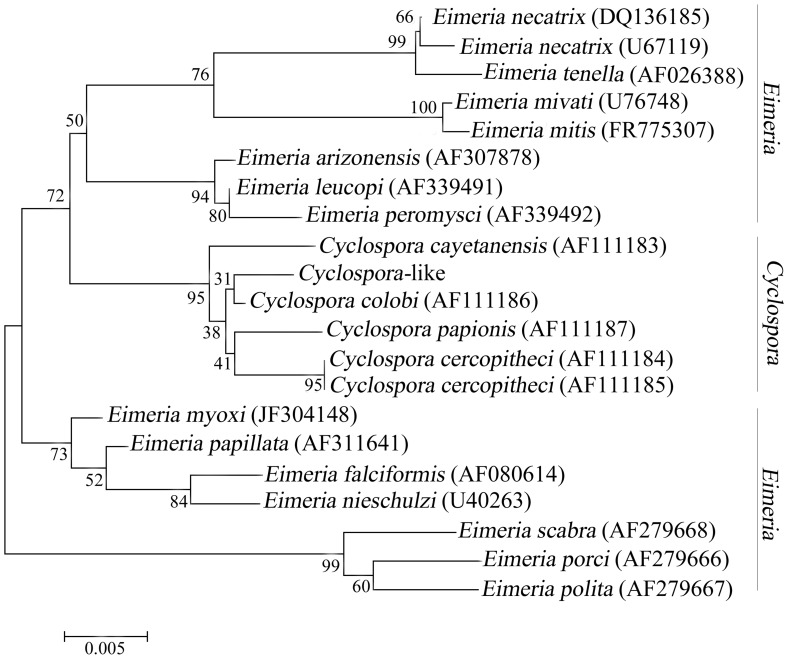
Phylogenetic relationships of *Cyclospora* isolates from golden snub-nosed monkey with *Cyclospora* spp. and *Eimeria* spp. Phylogenetic analysis was based on 18S rDNA sequences using neighbor–joining (NJ) method. The consensus tree was obtained after bootstrap analysis with 1000 replications, with values above 50% reported.

Then the phylogenetic relationships of some common parasites in the genera *Eimeria* and *Cyclospora* were re-constructed using NJ method based on partial 18S rDNA sequences (Figure 3). All of the *Cyclospora* species clustered together with high bootstrap value (95%). Within this clade, *Cyclospora*-like organism isolated from golden snub-nosed monkeys in the present study grouped with other monkey *Cyclospora* spp. and sistered to *C. colobi*. These results further confirmed that the *Cyclospora*-like organism isolated herein represents *C. colobi*.

## Discussion

In the present study, *C. colobi* was the only species found in the golden snub-nosed monkey within an observation period of one year. The common human species *C. cayetanensis* was not found. This finding was consistent with results of previous studies in other monkeys in Ethiopia [34] and Kenya [44]. These results may be explained by two reasons. Firstly, monkeys may be unlikely to be animal reservoirs of the human pathogen *C. cayetanensis*. Therefore, the transmission of *Cyclosopora* infection between non-human primates and human needs further study in the future. Another explanation is the limitation of the detection methods commonly used for the detection of *Cyclosopora* oocysts. The Sheater’s sugar flotation technique has a low sensitivity, and fecal samples with light infection intensity may not be detected.

From July 2011 to August 2012, only two *Cyclosopora*-positive samples were detected in July and August of 2011, respectively, which is different from study by Eberhard et al. [44] who reported that no marked seasonality was observed for *Cyclosopora* infection in African monkeys, despite the existence of distinct weather patterns in Kenya, East Africa. However, our results were consistent with findings of *C. cayet*anensis infection in humans in Henan province [15] where *C. cayetanensis* infection occurred in the hottest and most rainy months of the year. These results might indicate a seasonality of the infection, but may also relate to the low sensitivity of the Sheater’s sugar flotation technique used for screening the feces for detection of oocysts, or the small number of examined fecal samples (N = 71) due to the endangered nature of the golden snub-nosed monkey. In addition, the *Cyclospora* infection of golden monkeys could be transient due to environmental fecal contamination and immunity [13], [45], which suggested that fecal contamination might be an important mode of transmission for this parasite [46].

In conclusion, the present study isolated and characterized two *Cyclospora* isolates from the golden snub-nosed monkeys in Qinling Mountain in Shaanxi Province, Northwestern China. These two *Cyclospora* isolates represented *C. colobi* based on their morphological features and 18S rDNA sequences. This is the first report of *C. colobi* from the endangered golden snub-nosed monkeys. These results have important implications for transmission study and control of cyclosporiasis in humans and animals.
